# From Childhood Scars to Military Service: The Hidden Burden of Trauma

**DOI:** 10.1192/j.eurpsy.2026.11150

**Published:** 2026-07-31

**Authors:** A. Kammoun, A. Aissa, W. Ben Fleh, L. S. Chaibi

**Affiliations:** 1 Military Hospital of Tunis, Tunis; 2 Razi Hospital, Manouba, Tunisia

## Abstract

**Introduction:**

Childhood trauma is a well-established risk factor for the development of psychiatric disorders and maladaptive behaviors, including alcohol use. Military personnel are particularly vulnerable due to occupational stressors that may exacerbate the impact of early adverse experiences. Despite its relevance, the relationship between childhood trauma and alcohol consumption in military outpatients remains poorly understood.

**Objectives:**

The aim of this study was to examine the association between childhood trauma and alcohol use among military personnel receiving outpatient psychiatric care.

**Methods:**

A cross-sectional study was conducted from September to December 2024 at the Military Psychiatry Outpatient Unit of the Principal Military Teaching Hospital of Tunis. Tunisian military personnel followed for a psychiatric disorder for at least six months were included. Childhood trauma was assessed using the 28-item Childhood Trauma Questionnaire (CTQ), covering emotional, physical, and sexual abuse, as well as emotional and physical neglect. Alcohol use was evaluated with the 10-item Alcohol Use Disorders Identification Test (AUDIT), with a cutoff score ≥8 defining alcohol use disorder.

**Results:**

Participants with alcohol use disorders had significantly higher total CTQ scores (60.5 ± 12.9) compared to those without alcohol problems (50.5 ± 11.7; p = 0.003), indicating a higher burden of childhood trauma. Analysis of specific trauma domains revealed a significant association between physical abuse and alcohol consumption (p = 0.016). No significant associations were found for emotional abuse, sexual abuse, emotional neglect, or physical neglect (p = 0.399–0.699). Notably, the absence of reported emotional abuse limited the ability to assess its potential link with alcohol use in this sample

**Conclusions:**

Childhood physical abuse is a key risk factor for alcohol use disorders in military personnel, underscoring the need for trauma-informed assessment and early intervention in psychiatric care

**Disclosure of Interest:**

None Declared

